# Parent-child Communication, Social Norms, and the Development of Cyber Aggression in Early Adolescence

**DOI:** 10.1007/s10964-022-01625-1

**Published:** 2022-05-20

**Authors:** Anna Bullo, Peter J. Schulz

**Affiliations:** 1 Faculty of Communication, Culture and Society, Università della Svizzera italiana, Lugano, Switzerland; 2 Department of Communications and Media, Ewha Womans Univeristy, Seoul, South Korea

**Keywords:** Early adolescence, Parent-child communication, Cyber aggression, Social norms, Longitudinal

## Abstract

To understand the development of cyber aggression during adolescence, it is important to consider the temporal variability of its potential predictors. This study uses a four-wave survey to investigate how changes in peer norms, parental norms, and parental communication are associated with two-year trajectories of online peer aggression. The sample includes 1521 Swiss middle school students (M_age_ T1 = 11.54, SD = 0.40; 48% female). The results showed that over time a better parental communication quality and anti-aggression norms predicted lower rates and slower development of cyber aggression. Moreover, parental variables emerged as a quite stable deterrent of aggressive conduct. Although entrance into adolescence is characterized by the rise of peer influence, results from this study suggest that parents maintain an important protective role.

## Introduction

While online communication technologies offer various development and learning opportunities (Itō et al., 2009), they have also increased the possibility to contact and be contacted by people with less benign things on their minds. This represents an especially fertile ground for the perpetration of peer aggression. Indeed, although prevalence estimates tend to be highly heterogeneous across studies, recent data suggests that cyber-based aggression is a rather common behavior among children and adolescents all over the world, with prevalence rates ranging from 6 to 46% (Zhu et al., 2021). As the consequences of cyber aggression are as serious as those observed in offline settings—anxiety, depression, conduct problems and substance consumption are just some of the negative outcomes that cyber victims, and their aggressors also, may experience (Kowalski et al., 2014)—the interest in identifying the underlying mechanisms and preventive factors is strong. This study adopts a longitudinal approach to investigate how changes in peer norms, parental norms and parental communication are related to the development of cyber aggression among early adolescents.

### Parental and Peer Norms

Social norms have been defined as unwritten codes of conduct that are socially negotiated and understood through social interaction, and they include both perceptions of what others do (descriptive norms) and about what important others expect one to do (injunctive norms) (Chung & Rimal, 2016). According to the most prominent theories about social norms (Theory of Reasoned Action by Fishbein & Ajzen, 1975; Theory of Planned Behavior by Ajzen, 1991; Focus Theory of Normative Conduct by Cialdini et al., 1990; Social Norms Approach by Perkins & Berkowitz, 1986; Theory of Normative Social Behavior by Rimal and Real, 2005), individual conduct is commonly shaped by significant others’ behavior and expectations. The act of conforming—which is rarely the result of a conscious decision-making process—satisfies essential needs, such as the need of accuracy (Cialdini, 2008), coordination and social acceptance (Young, 2015). Transgressing social norms, on the other hand, can lead to social sanctions, including loss of status and ostracism (Bicchieri, 2005).

During childhood and adolescence, norms deriving from parents are considered a critical resource. The relationship between parents and their children has indeed an important socializing function: most of the habits, skills, values, and motives that permit children to become adults able to adequately function within the requirements of a society are learned through parent-child interactions (Maccoby, 1992). This pervasive and long-lasting impact is mainly attributable to the continued exposure of children to their parents’ normative messages (Kranstuber Horstman et al., 2016). Interestingly, these normative messages can be made explicit through the establishment of specific rules, but also learned through more implicit forms of communication, such as storytelling, conversing and modeling (Maccoby, 1992). In addition, although children will sometimes comply with parental expectations to avoid punishment (Hinduja & Patchin, 2013), they will do so at other times to seek affinity with and approval from their parents (Steinberg, 2001). The key role of parental norms is well illustrated by research on risky behaviors: previous studies have proven that norms of parents can be effective in deterring problematic conduct such as smoking (Hiemstra et al., 2017), alcohol drinking (Wood et al., 2004), unsafe sex practices (Jaccard & Dittus, 2000), as well as delinquency and aggression (Windle et al., 2010). While parental practices typically aim at diminishing risk taking (Elsaesser et al., 2017), it is important to note that sometimes they can also have a negative effect. Indeed, when parents set a bad example, their offspring is considered more at risk. For instance, children who perceive higher rates of parental conflict are more likely to act aggressively (Vu et al., 2016).

A further important influential figure in the life of children and adolescents is that of peers. The impact of norms deriving from friends and schoolmates is known to increase as children approach adolescence, as at this age they become highly susceptible to peer group acceptance and social status (Steinberg & Monahan, 2007). Because many adolescents are willing to adjust to peer behavior even when dangerous or unhealthy activities are involved (Brown & Anistranski, 2020), the role of peer norms is critical in the prevention of risky behaviors: past research indicates that the normative influence of peers is an important predictor of adolescent smoking (Perkins et al., 2019), alcohol drinking (Teunissen et al., 2012), risky driving (Simons-Morton et al., 2012) and unsafe sexual practices (Widman et al., 2016). For what concerns aggressiveness, findings from various studies show that the perception of peers behaving aggressively and supporting aggression leads to an increased likelihood of individual aggressive conduct (e.g., Busching & Krahé, 2015; Espelage et al., 2003; Kikas et al., 2009; Werner & Hill, 2010). Interestingly, although peers are often identified as a factor of risk (Veenstra & Dijkstra, 2011), research has also demonstrated that when their norms sustain prosocial behavior they have an important protective effect (Hofmann & Müller, 2018).

Because nowadays the use of online communication technologies is more and more widespread among children and adolescents (Smahel et al., 2020), some researchers have started to investigate whether social norms are related also to online behavior. Results from various studies suggest that anti-aggression norms deriving from parents and peers can indeed discourage engagement in detrimental activities such as cyberbullying and cyber aggression (e.g., Dang & Liu, 2020; Festl et al., 2014; Heirman & Walrave, 2012; Hinduja & Patchin, 2013). However, while these findings show that norms of parents and peers are relevant factors to consider in preventing online aggression, it should be acknowledged that important developmental questions remain unanswered. Most research on social norms is indeed cross-sectional in nature (Schulman et al., 2017), and even the few existing longitudinal studies have typically treated social norms as static constructs (Laninga-Wijnen et al., 2016). Considering the numerous behavioral and social changes that take place during childhood and adolescence, this constitutes an obvious limitation. Indeed, not only online aggressive behaviors have been reported to transform throughout adolescence years (Sumter et al., 2012), but social relations are known to change as well. As previously discussed, entrance into adolescence is characterized by the growing importance of peers (Steinberg & Monahan, 2007), a change that might challenge the normative influence of parents. In addition, with adolescence children become increasingly independent, a change that has profound effects on the relationship with their parents (Keijsers & Poulin, 2013) and might determine a decline in the effectiveness of parental norms (Wood et al., 2004). However, in the absence of longitudinal studies, it remains unclear whether peer and parental norms about aggression do actually change and, most importantly, whether normative changes are associated with modifications in perpetration of online peer aggression.

### Parent-child Communication Quality

An important consideration to make is that parental norms can be conveyed not only through explicit rules, but also through more indirect forms of communication, such as conversing and modeling. Because parent-child communication constitutes an important model on the basis of which children shape their interactions with peers (Kranstuber Horstman et al., 2016), it constitutes a further important predictor of children and adolescent aggressive behavior. Particularly, a positive family communication is considered a preventive factor for both traditional bullying (Griffin & Gross, 2004) and cyber aggression (Romero-Abrio et al., 2019). However, as with social norms, entrance into adolescence brings significant transformations for parent-child interactions and children’s attempt to assert their growing autonomy represents a considerable challenge to the maintenance of good parent-child communication (Keijsers & Poulin, 2013). This means that as kids get out of childhood and into adolescence the quality of parent-child interactions generally tends to worsen (De Los Reyes et al., 2015). Studies indeed report that during these years children normally perceive increased conflict with their parents, as well as lower family closeness and support (De Los Reyes et al., 2015). However, there is also evidence that communicative patterns tend to get better as soon as parents and their adolescent children no longer have to negotiate issues of autonomy and independence (De Goede et al., 2009). Interestingly, although these results indicate that the quality of parent-child communication should be considered as a dynamic variable, there is still little knowledge about its longitudinal association with online aggression (Camerini et al., 2020). In addition, it is important to consider that the quality of parent-child communication might interact with parental norms. A comprehensive explanation for this effect is offered by the parent-adolescent communication framework developed by Jaccard et al. (2002): according to this framework, social norms are among the immediate determinants of children’s behavioral intention that can be significantly influenced by parental communication. In addition, the framework identifies various aspects of communication that can alter key behavioral determinants (i.e., child’s attitudes, beliefs, affective and emotional reactions, self-efficacy, self-concept, and social norms) and sustain parents’ attempts to prevent undesired risky conduct. These aspects include characteristics of the message itself (e.g., message style and types of arguments), but also of the source, recipient, channel, and context of communication (Guilamo-Ramos et al., 2006). In the present research, the characteristics of the source—i.e., the parent—are of particular interest. As suggested by previous research, perceived expertise (i.e., child’s perception that the parent gives good advice and is a credible source of information), perceived trustworthiness (i.e., child’s perception that the parent is honest, trustworthy, and looking out for the best interests of the child) and perceived accessibility (i.e., child’s perception that the parent is available to talk and give advice) are three important dimensions of parent-child communication that permit to achieve the degree of affinity and desire for connection that are indispensable to obtain a behavioral change (Guilamo-Ramos et al., 2006). However, while theory of social norms deems that the relationship with the normative source is critical for norms conveyance (Lapinski & Rimal, 2005), whether parental norms and the quality of parental communication significantly interact over time remains an open question.

## Current Study

Different aspects of family relationship and practices have been proven fundamental in reducing the risk of in-person peer aggression, including peer norms, parental norms, and parent-child communication quality. Although in the last few decades research has started to examine the role of these factors also in online contexts, it is important to note that most studies do not account for their dynamic nature. As the onset of adolescence is marked by important changes in relationships with both parents and peers, this constitutes a major shortcoming. The main aim of the present longitudinal study is therefore to determine whether changes in aggressive behavior go hand in hand with normative and communicative ones. Based on theory of social norms, it was hypothesized that early adolescents who perceive peer norms that are more disapproving of aggression would experience both lower initial state and a less steep increase in their online aggressive behavior as compared to those who perceive more tolerant peer norms (Hypothesis 1). A similar effect was hypothesized for parental norms, so that adolescents’ perception of higher parental disapproval of aggression would be associated with both lower initial state and a less steep increase in online aggressive behavior (Hypothesis 2). Then, as the role of parents was expected to be more prevalent among younger children, it was further hypothesized that the effect of parental norms on the initial state of online aggression would be larger than that of peers (Hypotheses 3). Conversely, as the role of peers strengthens over time, it was predicted that the rate of change of online aggressive behavior would be more strongly associated with peer norms than with parental norms (Hypothesis 4). Finally, based on the communication framework of Jaccard et al. (2002), it was hypothesized that perceptions of a better parental communication quality would be related to both a lower initial state and a less steep increase of acts of online aggression (Hypothesis 5) and that it would have a significant moderating effect on the relation between parental norms and online aggression (Hypothesis 6).

## Methods

### Participants and Procedure

Data for this research are drawn from four waves of a six-waves panel survey conducted among middle school students in the Canton of Ticino, Switzerland. Ticino is the southernmost canton of Switzerland and the only one with Italian as the major language. The region is characterized by few densely populated urban centers and various valleys of a rural nature. The Canton is considered one of the most prosperous regions of Switzerland and Europe (Baechler et al., 2017). Cantonal statistics indicate that 75% of middle school students are Swiss, 13% Italian and less than 10% come from other European countries (DECS, 2019).

The survey was conducted with the approval and collaboration of local school authorities and involved a representative sample of students. Specifically, at the beginning of the study (September 2017), a random sample of 66% of all first-year public school classes in the five areas of the canton was drawn. Those private schools having more than two first-year classes were also invited to participate in the study and one of the three schools that corresponded to this criterion accepted the invitation. For participation in the research, one of the two first-year classes of this school was randomly selected. The research therefore included 101 classes randomly selected from 1 private and 35 public middle schools.

For the purposes of the present research, data collected on four different occasions were analyzed: November/December 2017 (T1), May/June 2018 (T2), November/December 2018 (T3) and May/June 2019 (T4). The observation time thus ranged from the beginning of the first grade to the end of the second grade (M_age_ = 12.29, SD = 0.69; Min_age T1_ = 10.71, Max_ageT4_ = 15.44). A total of 2052 students were invited to participate at T1. Of these 2052, 2022 participated at T1, 1879 participated at T2, 1896 at T3 and 1865 at T4.

Data were collected through self-administered paper-and-pencil questionnaires that were completed in class under the surveillance of a teacher. Data obtained from the four waves were matched thanks to a unique identifier associated with the student’s name to which only collaborating school staff had access for survey distribution purposes. To protect participants’ privacy, teachers were instructed to insert all completed questionnaires in an envelope and seal it in front of the students. In addition to authorization from the cantonal school authorities, parents of the students were informed about the study and about the possibility to exclude their children (passive consent). Less than 4% of the students (*N* = 75) did not participate because their parents objected. The remaining missing data are due to absences the day of data collection or students moving outside the Canton.

### Measures

#### Cyber peer aggression

Cyber aggression (or online aggression) is defined as an “intentional behavior aimed at harming another person or persons through computers, cell phones, and other electronic devices, and perceived as aversive by the victim” (Schoffstall & Cohen, 2011). Specifically, the perpetration of cyber aggression towards peers was measured on four occasions with 6 of the 11 items that the European Cyberbullying Intervention Project Questionnaire (ECIPQ; Del Rey et al., 2015) used to assess the perpetration of aggressive acts online. The selected items covered verbal aggression (e.g., “I said nasty things to someone or called them names using texts or online messages”), relational aggression (e.g., “I spread rumors about someone on the Internet”) and media-related aggression (e.g., “I posted embarrassing videos or pictures of someone online”). Excluded items concerned hacking skills, creating fake accounts, altering pictures/videos of others, or posting online personal information about someone. Participants were asked to report how often they had engaged in these actions in the previous six months, using a 4-point response scale that went from “Never” (1) to “Often” (4). Cronbach’s alpha was 0.70 at T1, 0.79 at T2, 0.83 at T3 and 0.83 at T4. The measurement invariance of online peer aggression was tested in a structural equation modeling framework using confirmatory factor analysis showing high strong and strict factorial invariance. Additional details have been reported elsewhere (Bullo & Schulz, 2022).

#### Perceived parental and peer norms

Perceived injunctive norms, namely the perceived acceptability of a behavior, have been commonly assessed by asking people to indicate how much other important individuals or groups are expected to react to a particular behavior (e.g., Compernolle, 2017; Joyal-Desmarais et al., 2019; Shin et al., 2015). Injunctive norms deriving from both parents and peers were therefore assessed by asking participants to evaluate how their parents and peers would react if they engaged in aggressive behaviors towards a schoolmate. For both peer and parental norms, four indicators covered both acts of traditional aggression (i.e., in-person insults and kicking or hitting) and online aggression (i.e., insults via communication technologies and sharing embarrassing pictures or videos). Answers were provided using a 6-point smiley face scale that went from an angry/sad face (1 = complete disapproval) to a happy face (6 = complete approval). Cronbach’s alpha for the items about peer norms was 0.90 at T1, 0.88 at T2, 0.92 at T3 and 0.905 at T4. Cronbach’s alpha for the four items about parental norms was 0.80 at T1, 0.82 at T2, 0.83 at T3 and 0.89 at T4.

#### Perceived quality of parental communication

At T2, T3 and T4 parental communication quality was assessed using a scale developed by Guilamo-Ramos et al. (2006) that includes the dimensions of perceived parental expertise, trustworthiness, and accessibility. While the original scale specifically focuses on the relationship between adolescents and their mothers, in the current study the items were modified so that they could apply to the parent they spent most time with. The scale consists of 9 items: three for perceived expertise (e.g., when I need good advice about something important, I go to my mother/father for help), three for trustworthiness (e.g., I can trust my mother/father when we talk), and three for accessibility (e.g., it is difficult for my mother/father and me to find time to talk). The items were rated on a 5-point scale that went from “Totally disagree” (1) to “Totally agree” (5). Cronbach’s alpha for this scale was 0.81 at T2, 0.83 at T3 and 0.87 at T4. Because this scale was not available for T1, the quality of parental communication was measured using four items that investigate children’s perceptions of their parents’ perspective taking (i.e., if parents try to understand their point of view, if their parents understand what they are going through when talking about important matters, if it is easy for their parents to put themselves in their place and if their parents want to understand their side of things). These four items were rated on a 5-point scale that went from “Never” (1) to “Always” (5). Although the scale had been previously used in another longitudinal study conducted among middle school students and shown acceptable reliability (Marciano et al., 2020), in this study the Cronbach’s alpha value was 0.57.

#### Age

The exact age of participants at each data collection was computed from their birthdate.

### Analytic Plan

A total of 2046 students participated at least in one of the four waves. Cases with a possible administrative error in sample management were removed (*N* = 355) as were cases in which the answers were considered unreliable (*N* = 55). Then, the sample was further narrowed by selecting for the analyses only those subjects who participated in at least three waves (*N* = 1521).

In the analytic sample, missing data related to the variables of interest were rather limited (<5%). Little’s missing completely at random test indicated that data in the scales included in the analyses were missing at random (χ^2^ = 3021, *N* = 1521) = 3009.81, *p* = 0.554. To impute the remaining missing values, a Bayesian regression imputation method and predictive mean matching models were used. Finally, the presence of outliers was checked using Mahalanobis and Cook’s distance and leverage methods. As no outliers were identified, the final analytical sample remained of 1521 participants.

To analyze longitudinal data about cyber aggression and the other related factors, growth models within a multilevel modeling approach were used (cf. Singer & Willet, 2003). A multilevel model for change is indicated because it permits to investigate both change within-persons and between-persons. More specifically, the analysis of the within-person change (Level 1) concerns the individual development that each subject experiences over time and that is attributable to a personal combination of different influence factors. On the other hand, the change between-subjects is related to influence factors that are common to groups of subjects in a sample. In the present study the interest is in how the changes in cyber aggression differ between subjects according to different levels of perceived peer norms, parental norms and parental communication quality. The two-level hierarchical models were estimated using a Maximum Likelihood method in SPSS Statistics 25. The first step of the analysis involves the estimate of an unconditional mean model. This first model estimates both a fixed effect and a random effect, provides an estimate of the grand mean of cyber aggression, and indicates whether there is systematic variation worth exploring. In addition, the model specifies how much of this variability is due to between-person differences and how much lies within individuals. The unconditional mean model represents a benchmark to compare model fit measures of alternative models, as it does not include any influence factors at either Level 1 or Level 2. Because the estimates of the variance components indicated that there were significant differences in cyber aggression scores to be explained, both within and between subjects, the analysis continued with the estimation of an unconditional growth model. The unconditional growth model is characterized by the addition of the parameter of time (in this case participants’ age minus mean age at T1) as a possible influence factor. The inclusion of a time factor permits to assess the baseline amount of change in cyber aggression. This model is especially useful to answer hypotheses about the effect of time, as it indicates how much of the variance within and between subjects can be attributed to the ageing of subjects, but also represents a baseline for evaluating subsequent models. Indeed, to address the hypotheses, a series of conditional models were examined. This procedure allows to determine the relative contribution of each new variable on top of those factors that were already considered in the earlier models. More specifically, the peer norm was first added to the analysis, then the parental norm, the parental communication quality and finally the interaction between parental norms and parental communication quality. All these variables were mean- centered prior to the analysis and tested as possible influence factors of both initial status and change: the intercept represents each subjects’ average level of cyber aggression, while the coefficient on time indicates the six-month increase.

Because the dependent variable resulted to be highly skewed—a common problem with self-reports of undesirable behaviors such as cyber aggression—the analyses were conducted also with the MLR estimator in MPlus, which is less sensitive to non-normally distributed data. In addition, to reduce the skewness, the analyses were also run in STATA using log-transformed data. In both cases, the results of the analyses show only small differences in coefficients, while the significance of the tested models remained the same. For greater clarity in the presentation of findings only data stemming from the analyses conducted with SPSS are reported. These findings are based on non-transformed data and provided more information about between- and within-variance.

## Results

The 1521 subjects who participated in at least three waves (74%) had a mean age of 11.54 at T1 (*SD* = 0.40) and 13.04 (*SD* = 0.40) at T4, and about 48% of them are female. The descriptive statistics for cyber aggression, parental communication quality, peer and parental norms are shown in Table 1. A first consideration derived from this table is that the overall level of self-reported cyber aggression is very low, being just above the minimum value. However, it can be noted that the scores tend to grow between the first and the last assessment, with only a slight drop between the second and third and waves. Data about peer norms show an opposite trend: as they grow older, children generally appear to perceive less disapproval of aggressive behaviors from their schoolmates. Considering that complete approval corresponds to a score of 1, it is however important to note that, with a minimum score of 4.45 at T4, the norm deriving from peers mostly remains opposed to aggression. For what concerns parental norms, the average values are quite high, indicating that children are generally aware that they would disappoint their parents if they were to engage in aggressive behaviors. However, in this case mean scores do not show a linear pattern: while the perceived tolerance for aggression decreases between T1 and T2 and between T3 and T4, it increases between T2 and T3. Finally, descriptive results about parental communication show that the perceived quality tends to remain quite good, with scores that settle around 4 out of 5. The lower score observed at T1 could be attributable to the fact that a different scale was used. From T2 to T4, where the measurement was the same, a small decrease in mean values can be observed, indicating a slight deterioration in the perceived quality of interactions with parents.

**Table 1:** Descriptive Statistics: Longitudinal assessment of cyber aggression, peer norms, parental norms and parental communication quality

	Waves			
	T1	T2	T3	T4
*Cyber aggression*				
*N*	1521	1521	1521	1521
Mean (SE)	1.11 (0.01)	1.21 (0.01)	1.18 (0.01)	1.26 (0.01)
Skewness (SE)	3.61 (0.06)	2.68 (0.06)	3.90 (0.06)	2.41 (0.06)
Kurtosis (SE)	17.33 (0.13)	10.13 (0.13)	21.34 (0.13)	7.04 (0.13)
*Peer injunctive norm*				
*N*	1521	1521	1521	1521
Mean (SE)	4.78 (0.03)	4.70 (0.03)	4.59 (0.03)	4.45 (0.03)
Skewness (SE)	−0.94 (0.06)	−1.10 (0.06)	−1.03 (0.06)	−0.91 (0.06)
Kurtosis (SE)	0.99 (0.13)	1.81 (0.13)	1.41 (0.13)	1.26 (0.13)
*Parental injunctive norm*				
*N*	1521	1521	1521	1521
Mean (SE)	5.39 (0.02)	5.58 (0.01)	5.37 (0.02)	5.49 (0.02)
Skewness (SE)	−1.01 (0.06)	−1.95 (0.06)	−1.76 (0.06)	−2.11 (0.06)
Kurtosis (SE)	0.90 (0.13)	6.54 (0.13)	6.26 (0.13)	7.21 (0.13)
*Parental communication quality*^a^				
*N*	1521	1521	1521	1521
Mean (SE)	3.92 (0.02)	4.37(0.02)	4.22 (0.02)	4.21 (0.02)
Skewness (SE)	−0.45 (0.06)	−1.11(0.06)	−0.87 (0.06)	−1.12 (0.06)
Kurtosis (SE)	−0.29 (0.13)	1.20 (0.13)	0.53 (0.13)	1.30 (0.13)

^a^The measure of parental communication quality at T1 differs from that at T2, T3 and T4

Table 2 reports the results of growth models predicting cyber aggression and the Goodness of fit related to all the tested models. Model A represents the unconditional mean model, which estimates the grand mean of cyber aggression across all individuals and all waves. Model B is the unconditional growth model, which includes the variable of time. The remaining models are all conditional models which progressively add variables as potential influential factors of both initial status and change in cyber aggression: Model C introduces peer norms, Model D parental norms, Model E parental communication quality and Model F the interaction between parental communication and parental norms. Every additional predicting variable contribute to improve the overall Goodness of Fit. Moreover, they help to explain both within- and between- person variance. For what concerns the within-person variance, it can be noted how values progressively decrease: this means that individual variance is related to the predicting variables introduced in the models. Regarding the between-person variance, it is interesting to note that not only values tend to shrink, but also that in model E the value related to the initial status becomes non-significant. This means that differences between subjects in initial levels of cyber aggression can be completely explained by differences in perceived norms and parental communication. Because the interaction between parental norms and communication quality is not significant and no substantial differences emerge in the estimated effects, Model E is considered the final model.

**Table 2:** Results of fitting a multilevel model for change to the cyber aggression data: fixed effects, variance components and Goodness of fit

		Model A	Model B	Model C	Model D	Model E	Model F
Initial status	Intercept	1.19*** (0.007)	1.13*** (0.006)	1.13*** (0.006)	1.12*** (0.006)	1.13*** (0.006)	1.13*** (0.006)
	Peer norm			−0.04*** (0.006)	−0.03*** (0.071)	−0.02* (0.007)	−0.02* (0.007)
	Parental norm				−0.06*** (0.011)	−0.05*** (0.012)	−0.05*** (0.012)
	Parental com. quality					−0.04*** (0.008)	−0.04*** (0.008)
	Parental norm*parental com. quality						0.01 ns (0.013)
Rate of change	Intercept		0.08*** (0.006)	0.08*** (0.006)	0.08*** (0.006)	0.08*** (0.006)	0.08*** (0.006)
	Peer norm			−0.02*** (0.006)	−0.02* (0.006)	−0.02* (0.006)	−0.02* (0.006)
	Parental norm				−0.03* (0.010)	−0.03* (0.011)	−0.02* (0.011)
	Parental com. quality					−0.02* (0.008)	−0.02* (0.008)
	Parental norm*parental com. quality						0.01 ns (0.013)
Level 1	Within-person	0.086*** (0.002)	0.066*** (0.001)	0.055*** (0.001)	0.050*** (0.001)	0.052*** (0.004)	0.051*** (0.003)
Level 2	In initial status	0.045*** (0.002)	0.019*** (0.002)	0.011*** (0.002)	0.011*** (0.002)	0.012 ns (0.010)	0.012 ns (0.006)
	In rate of change		0.016*** (0.002)	0.016*** (0.002)	0.015*** (0.002)	0.013* (0.004)	0.013*** (0.003)
	Co-variance		0.018*** (0.002)	0.011*** (0.002)	0.009* (0.002)	0.009*** (0.002)	0.009*** (0.002)
		Model A	Model B	Model C	Model D	Model E	Model F
Deviance		4057.32	3601.77	3066.04	2663.19	2483.11	2495.76
AIC		4061.32	3609.77	3080.04	2685.19	2515.20	2527.76
BIC		4074.75	3636.62	3127.03	2759.03	2622.50	2635.15

*Note:* Cells show the estimates and their standard deviations in brackets

*p < 0.05 **p < 0.01 ***p < 0.001

Data obtained for the unconditional growth model (Model B) indicate that engagement in cyber aggression significantly grows by 0.08 (*SE* = 0.006) points every six months. The same result was estimated also for the final model (Model E). The increase in cyber aggression over the four waves is therefore of 0.32 points (11%). Then, results from Model C indicate that children who reported higher levels of peer disapproval of aggression were significantly less likely to engage in cyber aggression (−0.04, *SE* = 0.006). A slightly lower effect (−0.02, *SE* = 0.007) is estimated when taking into account the impact of parental variables. For what concerns the rate of change, results indicate that peer norms have a significant effect also on the speed with which cyber aggression develops (−0.02, *SE* = 0.006). The first hypothesis (H1) was therefore confirmed.

Data from Model D show that subjects who perceived higher parental disapproval of peer aggression were also more likely to report lower involvement in cyber aggression (−0.06, *SE* = 0.071). The estimate is similar and significant also in the final model (−0.05, *SE* = 0.012). The association of parental norms with cyber aggression was significant also for the rate of change: the increase in cyber aggression was reduced by 0.03 (*SE* = 0.010) points every six months. The second hypothesis (H2) was therefore confirmed as well.

Results obtained for Model E indicate that adolescents who reported higher levels of parental communication quality were significantly less likely to report engagement in cyber aggression (−0.04, *SE* = 0.08). The effect of parental communication on the rate of change in cyber aggression was also significant (−0.02, *SE* = 0.008). The fifth hypothesis (H5) is therefore confirmed.

Model F tested the effect of the interaction between parental norms and parental communication quality; however, results are not significant, neither for the initial state nor for the rate of change. The sixth hypothesis (H6) was therefore not confirmed.

For what concerns the relative impact of peers and parents on the development of cyber aggression, the estimated effect of parental norms tends to be larger than that of peer norms, especially for the initial state (−0.05 and −0.02, respectively). For the rate of change the difference is smaller, but the estimated effect of parental norms (−0.03) remains higher than that of peers (−0.02). The third hypothesis (H3) was therefore confirmed, while the fourth (H4) was not.

## Discussion

Cyber aggression is a contemporary phenomenon that can have serious repercussions on the well-being of children and adolescents (Kowalski et al., 2014). While entrance into adolescence is known to be a period of important social changes, few studies have investigated the role of peer and parental influences from a longitudinal perspective. Indeed, while previous research highlights the important preventive role of parents and warns against the possible negative influence of peers, no study has explored whether the development of aggressive behavior online is related to changes in normative messages from these reference figures. In addition, while the theory of social norms posits that the effects of social norms are stronger when the relationship with the normative source is good (Lapinski & Rimal, 2005), there is still few research about whether the quality of parent-child communication can reinforce the impact of parental norms in deterring problematic behaviors. To help fill these knowledge gaps, the present study drew from both social norms theory and the parent-child communication framework of Jaccard et al. (2002) and examined how changes in peer norms, parental norms and parental communication quality are associated with change in online aggressive behavior.

The first step in the analysis was to determine whether changes in online aggressive behavior actually occurred in the sample. Although there are studies revealing that cyber aggression happens already among elementary school children (Kowalski et al., 2014), previous research suggests that acts of cyber aggression increase during early adolescence (Barlett & Chamberlin, 2017). Interestingly, while obtained results indicate that, during the first two years of middle school, cyber aggression remains a quite rare phenomenon, the existence of a growing path was confirmed. This increase might be partly explained by a larger use of online technologies (Suter et al., 2018), but also to changes in normative messages.

The first normative source examined was that of peers. As hypothesized, the growth models reveal that peer norms are significantly associated with peer aggression initial status and rate of change. This means that perceived peer approval of aggression is generally associated with higher rates of involvement in online aggression and that the pace of growth over time is the higher for both those perceiving a higher peer tolerance of aggression. This result is in line with previous research, which agrees that peers play an important role during the teenage years and that group behavior and expectations—whether real or perceived - can influence individual conduct (e.g., Dang & Liu, 2020; Festl et al., 2014; Heirman & Walrave, 2012). The influence of peers can be considered problematic when they share norms that support or condone risky behaviors: although results from this study show that peers are mostly expected to disapprove acts of aggression, it is interesting to note that over time this perception changes, shifting towards a slightly greater tolerance. As the developments of cyber aggression and peer norms are related, the consequences might become particularly serious if the observed trend persists in the following years.

Transformations in peer norms about risky conduct has already been reported by previous research, showing how adolescents perceive that their peers become progressively more accepting of substance use and other problematic behaviors (e.g., Duan et al., 2009; Meisel & Colder, 2019; Pedersen et al., 2013; Weaver et al., 2011). However, less is known about what happens to parental norms and to the perceptions that children have of them. Because adolescence is typically thought as a period of transgression, parents might be led to reinforce their normative messages, resulting in stricter perceived norms. Conversely, the fear that their child might engage in dangerous activities might render certain risky behaviors comparatively less serious in the eyes of the parent. For instance, as smoking a cigarette might be seen as less serious than taking drugs, and trying an alcoholic drink might be perceived as less problematic than getting completely drunk, insulting someone online could be seen as less problematic than assaulting someone. At the same time, during adolescence the experience of children with risky conduct increases, and this might lead to a correction of perceived norms that previously were based on mere guessing. For example, a child might be caught sharing an embarrassing picture of a friend and find out that their parents are not as upset as he or she had expected. Interestingly, descriptive data from the sample do not reveal an evident increasing or declining pattern for perceived parental norms, meaning that the latter tend to remain quite stable, at least during the very beginning of adolescence. However, what the results show is that children who perceive more disapproval of aggression from their parents tend to report lower involvement in online aggressive behaviors, and that the development of cyber aggression is more rapid for those children whose parents are perceived as more tolerant of violence. Because the present study focuses on a sample of early adolescents, the fact that parental norms are significantly related to child’s conduct is not at all surprising: at this age, parents are expected to still have a say in the life of their children (DeVore & Ginsburg, 2005). Even the fact that the effect of parental norms on the initial status is stronger than that of peers is not so unexpected: while the role of the latter is known to grow with the beginning of adolescence, it does not mean that peer norms are immediately stronger than those of parents. Indeed, there is evidence indicating that peer influence usually rises from late childhood and reaches its peak around the age of 14 (Brown & Anistranski, 2020). This means that the influential role of peers could catch up or surpass that of parents only at a later stage.

While findings from this study confirm that both peer and parents have the potential to counter or foster the spread of cyber aggression, what did not meet the expectations is the relative contribution of peers and parental norms on the rate of change in aggression. Because the effect of peer norms was expected to increase over time, it was hypothesized that their impact on the rate of change would be higher than that of parental norms. However, results show that the effect of peer and parental norms is almost the same, with the latter being even slightly more impactful. Again, this might be imputed to the very young age of the sample: the fact that the values estimated by growth models are almost equivalent might indicate that peer norms have started to become more relevant. However, while they come close in importance to the norms of parents, they have not reached their peak yet.

Then, based on the parent-child communication framework of Jaccard et al. (2002), the role of parental communication quality was also explored. In line with previous literature, findings from this study show that those children who perceive a better parental communication (in this case characterized by higher perceived expertise, trustworthiness, and availability) are less involved in cyber aggression. In addition, it was found that the presence of higher parent-child communication quality was associated with a less steep increase in cyber aggression. This finding is highly relevant because it shows that it is not only important what parents say, but also how they say it. Because the theory of social norms posits that the conveyance of behavioral norms passes through the relationship with the normative source (Lapinski & Rimal, 2005) a significant interaction between parental norms and parent-child communication quality was also hypothesized. However, obtained results indicate that while both concepts are significantly associated with cyber aggression, their interaction is not significant, meaning that the impact of parental norms is not stronger for those children who perceive a better parental communication. Nonetheless, when taken individually, the quality of parent-child communication between remains an important preventive factor to consider: although adolescents, as compared to children, tend to report less family closeness and support, less communication with their parents, and increased family conflict (De Los Reyes et al., 2015), it does not necessarily mean that their relationship with their parents becomes bad. Indeed, findings from the present research indicate that the quality of communication remained generally good over time. This result is in line with the fact that despite some inevitable divergences, most adolescents experience positive relationships with their family (Smetana et al., 2006).

To sum up, results from this study suggest that, as role models, parents can make a significant difference in their child’s online aggressive behavior. Interventions aiming at counteracting the development of online aggression during early adolescence might particularly benefit from knowing that parental norms and communication tend to be quite stable deterrents of aggressive conduct. In addition, it can also be useful for parents to know that their practices remain effective, despite the rise of peer influences.

Despite the useful insights offered by the current study, some limitations must be acknowledged. A first limit concerns the assessment of online aggression: as indicated in the method section, some of the original items of the ECIPQ scale (Del Rey et al., 2015) were excluded from the surveys. Since the research from which data were drawn does not exclusively investigate online aggression but also other risky behaviors, the motivation for this exclusion is due to space limitations. This decision allowed to focus on the dimensions of cyber aggression that were deemed more relevant to a sample of early adolescents, but it also caused some dimensions (i.e., media-related violence) to be underrepresented. A further issue concerns the use of a four-point scale, which is not very sensitive: a different sensitivity of the response scales used may have affected the results relating to over time changes. Then, it cannot be excluded that the low rates of cyber aggression observed across all waves might be due to a social desirability bias, especially because self-reported measures were used.

A second limitation in operationalization is related to measurement of communication quality. Indeed, as reported in the method section, at T1 the measurement of communication quality of Guilamo-Ramos et al. (2006) was not available. The quality of parent-child interactions was therefore assessed using a different scale consisting of four items. Unfortunately, in addition to being different, the scale was found to be less reliable. Although in both cases a 5-point answer scale was adopted, descriptive data indicate that the score obtained at T1 was evidently lower as compared to those of T2, T3 and T4. Because different items were used, it is impossible to determine whether the quality of communication was indeed perceived as worse or if this was an effect of the scale.

A further limitation concerns the time frame of the study. According to Singer and Willet, 2003, four waves is an adequate number of waves to conduct a multilevel model for change. However, because the measurement occasions took place at six-months intervals, it is true that the observed change is limited to only one year and a half. While this period of time corresponds to an important phase in the life of children (i.e., the beginning of middle school), a longer time frame could have provided a more accurate picture.

Then, it is important to note that, despite the longitudinal nature of the study, the analyses conducted do not allow causal conclusions to be drawn. While the theory of social norms and the framework of Jaccard et al. (2002) posit that social norms and parental communication are the cause of subsequent child behavior, it is important to note that children’s engagement in online aggression might contribute to alter parents’ norms and communicative practices as well. In addition, because all forms of peer aggression are strictly interrelated (Camerini et al., 2020), it would have been interesting to include engagement in traditional forms of aggression and victimization experiences as control variables.

Finally, as it is well known that differences between males and females increase as they enter adolescence (Blakemore et al., 2010), it might have been interesting to investigate the role of gender. Findings from a meta-analysis on cyberbullying indeed indicate that girls usually engage in relational cyber aggression at younger age, while boys increase these behaviors only during mid- and late adolescence (Barlett & Coyne, 2014). While this suggests that gender might determine separate developmental trajectories, results from the meta-analysis also indicate that around the age of 11 boys and girls appear to be equally involved in online aggression. For the sake of simplicity, the analyses were conducted without examining the role of gender. However, it should be acknowledged that gender might play a role also with regard to parent-child communication: a large body of research indeed reports that the communication between parents and girls is generally more open than between parents and boys (Racz & McMahon, 2011), and that throughout adolescence it follows distinct developmental patterns (Keijsers & Poulin, 2013). Future research could therefore examine whether the impact of parental communication on cyber aggression is moderated by gender.

In addition, further applications of the communication framework of Jaccard et al. (2002) in relation to aggressive behaviors online are recommended. While in this study the focus was on parental communication and social norms, there are still other important determinants to investigate. For instance, it would be interesting to test whether parental communication quality affect children’s self-concept and if this self-perception is related to engagement in cyber aggression. Finally, because data for the present study covered only one year and a half, future research should observe cyber aggression and social norms also for a longer period or among samples of different age.

## Conclusion

Although various studies on cyber aggression have proven the determinant role of both parents and peer, longitudinal evidence concerning the association of parental and peer factors with this problematic behavior was lacking. The present study aimed at filling this gap by investigating how changes in peer norms, parental norms, and parent-child communication quality are related to the development of cyber-based peer aggression during early adolescence. As predicted by the theory of social norms and by the communication framework of Jaccard et al. (2002), results from growth models indicated that over time the quality of parental communication and perceived norms were associated with both the initial state and rate of change in cyber aggression. More specifically, participants who reported a better interaction with their parents and those who perceived that their parents and peers would be more disapproving of aggression, were also those who reported less frequent cyber aggression perpetration as well as a less steep increase in such behavior. Interestingly, the association of online aggression with parental norms and communication was slightly stronger than that with peer norms, suggesting that during early adolescence the role of parents remains important. While it was hypothesized that the quality of parental communication could have moderated the effect of parental norms on cyber aggression, the analyses revealed that the interaction between these two variables is not significant. To better prevent the development of aggressive behaviors online, it is recommended that interventions conducted among early adolescents do not underestimate the role of parents in favor of peers. To understand whether they remain key figures even in mid- and late adolescence, further longitudinal research is needed.
